# Sensor and Component Fault Detection and Diagnosis for Hydraulic Machinery Integrating LSTM Autoencoder Detector and Diagnostic Classifiers

**DOI:** 10.3390/s21020433

**Published:** 2021-01-09

**Authors:** Ahlam Mallak, Madjid Fathi

**Affiliations:** Department of Electrical Engineering and Computer Science, Knowledge-Based Systems and Knowledge Management, University of Siegen, 57076 Siegen, Germany; fathi@informatik.uni-siegen.de

**Keywords:** deep learning, LSTM autoencoder, supervised learning, hydraulic test rig, sensor faults, component faults

## Abstract

Anomaly occurrences in hydraulic machinery might lead to massive system shut down, jeopardizing the safety of the machinery and its surrounding human operator(s) and environment, and the severe economic implications following the faults and their associated damage. Hydraulics are mostly placed in ruthless environments, where they are consistently vulnerable to many faults. Hence, not only are the machines and their components prone to anomalies, but also the sensors attached to them, which monitor and report their health and behavioral changes. In this work, a comprehensive applicational analysis of anomalies in hydraulic systems extracted from a hydraulic test rig was thoroughly achieved. First, we provided a combination of a new architecture of LSTM autoencoders and supervised machine and deep learning methodologies, to perform two separate stages of fault detection and diagnosis. The two phases were condensed by—the detection phase using the LSTM autoencoder. Followed by the fault diagnosis phase represented by the classification schema. The previously mentioned framework was applied to both component and sensor faults in hydraulic systems, deployed in the form of two in-depth applicational experiments. Moreover, a thorough literature review of related work from the past decade, for autoencoders related fault detection and diagnosis in hydraulic systems, was successfully conducted in this study.

## 1. Introduction

Mechanical machines are considered a vital part of the industrial operation. Hence, they play a tremendous role in the production and manufacturing processes. Due to their major importance in the production line, they are usually placed in tough locations and tough environments, which make them susceptive to the occurrence of various faults and malfunctions. Faults in complex sensor systems can be defined as unexpected events that might occur at a certain point of time, which might trigger bigger events or a series of other unexpected events. According to Isermann and Balle [1], faults are defined as an unauthorized or allowed deviation of what is declared as normality of a defined system.

Nowadays, industrial applications are getting more complicated and scalable than ever, which contributed tremendously to the complexity of fault detection in those systems, and also made those tasks quite challenging [2]. In the literature, faults can be classified into three main categories, based on the location of the fault itself in the containing system, such as sensor faults, actuator faults, and component faults [3].

The study in [4] indicated that 70–90% of the incidents associated to industrial operations are caused by human workers or operators. Consequently, the need for computer-aided diagnosis emerged, to ensure highly accurate fault detection, prediction, and diagnosis of systems with extreme complexities. Moreover, computer-aided diagnosis might also contribute to the speed and precision of the recovery actions deployment required following fault appearance. The main goal of automated fault detection is to accurately capture the anomalies as soon as they manifest, to ensure deploying the necessary maintenance procedures, and to dodge economical, humanitarian, and environmental tragedies. Creating a solid fault detection and diagnosis systems contributes to reducing risk and providing safety to human operators and the environment. They also play a major role in cutting down costs related to unnecessary maintenance. Thereafter, fault detection and diagnosis in mechanical devices placed in complex systems like the industry is always a hot research topic.

Automated Fault Detection and Diagnosis (FDD) [5] algorithms and systems are usually dependent on the training and analysis of datasets, in which they are extracted from numerous sensors attached to the industrial equipment and its components. Those sensors continuously send essential signals to monitor each component of the mechanical machine. In other words, sensors’ readings are most often the modalities, or the source of row data associated with automated FDD systems. The health of these sensors is the key to monitor those components properly, which leads to accurate component diagnosis results. Although these sensors are substantial for computer-aided diagnosis, they are mostly ridiculously cheap and perform under extreme environmental conditions as they are attached to the machinery device. Therefore, sensors in mechanical machines are prone to malfunctions and variety of faults themselves. Sensor faults are the faults represented by the sensors and their readings. Usually, these faults are noticed when the sensors produce incorrect readings, due to a physical fault in the sensor itself, broken wires or a malfunction in the communication channels between the sensors and the controlling unit. A change in the sensor’s reading could also be an indicator (symptom) of a component or system fault. There are several existing classifications and categorical descriptions of sensor faults. The most sound and interesting one, is the comprehensive study conducted in 2009 [6], where an extensive approach was taken to provide a clear definition of each fault, their potential cause(s), the observed duration of each fault in time-series datasets, and the effect each fault carries on the sensed data. Smart FDD systems should monitor both the mechanical devices and their components, as well as the sensors’ health status of those who are responsible for reporting the health indications of mechanical components. Hence, it is undeniably important to establish sensor FDD systems along with the component FDD ones, when monitoring industrial operations.

One of the most essential mechanical equipment for industrial processes are hydraulic systems [7]. The hydraulic system’s data applied to this study was gathered from a hydraulic test rig. The applied dataset [8,9,10] represents real measurements of multivariate, time-series sensors, placed in a hydraulic test rig. The purpose intended for the data collection was to monitor and assess the hydraulic system health condition. A test rig could be defined as a piece of mechanical device that is mainly utilized to assess, evaluate, and test the capacities and performance of other mechanical machines, or just certain components of these machines. Test rigs can be called by various terminologies, including test bench or test pay, and testing station. Test rigs are common in a wide range of industrial fields, from hydraulic systems to aerospace. They have a vast scope of testing methods, and analytical parameters, such as, manual, cyclical, brake, and burst testing.

Hydraulic systems obtained from a hydraulic test rig were the focus of this study, due to their importance and limited FDD resources in the past decade, in which a comprehensive FDD system of both component and sensor faults was included.

Recently, an effective FDD framework is trending, which evolves around preforming the detection and diagnosis phases separately, to ensure detecting rare fault occurrences in various systems. The first phase is the detection, where it is often represented by a healthy signal reconstructed schema, using different Machine Learning (ML) and Deep Learning (DL) methodologies, i.e., autoencoders [11]. The autoencoder is trained using the fully efficient input of the dataset, which represents the healthy form of the training data. Eventually, the autoencoding model should be able to reconstruct the healthy version of the input data at any given point of time. Comparing the reconstructed healthy signal with the given one provides an indication of any fault. The more the given signal is identical to the healthy reconstructed one, the more likely that it is a healthy signal and lacks the presence of anomalies, and vice versa. Meanwhile, in the fault diagnosis phase, the faults or deviations captured in the first phase (detection phase), are then used to train a certain ML or DL classification model.

### Our Contribution

In this work, a comprehensive FDD approach for hydraulic systems is proposed. where an additional step is added in advance to the diagnosis, using the classification phase, to overcome the weaknesses of supervised diagnostic approaches in capturing rare and other faults existing beyond. To overcome this challenge, in this section the detection and diagnosis phases are performed separately. Where the detection phase is done by applying a Long Short-Term Memory (LSTM) [12] autoencoder to detect rare and unprecedented faults. This is followed by the diagnosis phase, which uses the ML and DL classifiers to analyze the nature of the captured faults in phase one.

This approach already exists in the literature. However, our work is beyond the state-of-the-art, due to the following. (1) It is the first study to apply this schema to hydraulic test rigs data. (2) This schema was applied to both sensor and component faults in a hydraulic test rig, in two separate thorough experiments. (3) In the detection phase represented by the LSTM autoencoder, we presented a new criterion to calculate the deviation between the predicted signal and the input one, which proved to be more effective than the traditional method in computing more accurate diagnostic thresholds. (4) In the fault diagnosis phase proposed by the classification, we provided a full comparison results between numerous ML and DL classifiers of different functionality and techniques. (5) In the same phase, we also provided a behavior analysis of each ML and DL classifiers used in the diagnosis phase with a bunch of time-domain feature selection methods, to help further research in the future, to map each classifier with their best or least suitable time-domain features to achieve either component or sensor FDD in hydraulic systems. (6) In this work, a comprehensive literature review for FDD in hydraulic systems, based on autoencoders is thoroughly presented.

The rest of paper is as follows. In Section 2, an in-depth analysis of the related work is presented, where a list of FDD research in hydraulic systems, based on autoencoding techniques for the past decade, is discussed. Section 3 explains the overview of the FDD system utilized in this work. In Section 4, the experimental results are showcased and analyzed in two main experiments, where one is applied on injected sensor faults, while the other is conducted to achieve FDD for component faults in hydraulic test rigs. Finally, the discussion, conclusion, and future work is discussed in Section 5.

## 2. Related Work

### Autoencoder Approaches for FDD in Hydraulic Machinery

The work in [13] shows a combined approach to achieve component fault detection and diagnosis of rare events occurring in chemical factories. The proposed method joins LSTM autoencoder as the detection phase, followed by the diagnosis phase using the LSTM classifier. This approach was used to detect and diagnose faults of the Tennessee Eastman benchmark [14], which represent a dataset extracted from a simulator of actual chemical processes that includes various components—reactors, condensers, vapor-liquids, etc. In the detection phase, the sequence comparison between the reconstructed sequence and the given one is achieved by applying the traditional signal difference. In the diagnosis phase, no feature selection or extraction approach is used prior to the classification using the LSTM classifiers. Moreover, a solo comparison to Convolutional Neural Network (CNN) [15] was made, but no comparisons with other DL or ML classifiers were conducted. Lu et al. [16] introduced a novel autoencoder called Stacked Denoised Autoencoder (SDA) that was used to detect component faults in rotary machineries. The method was applied to a dataset extracted from a physical simulation of a bearing test-rig. SDA implicitly feature engineer the data, which was compared to Principal Component Analysis (PCA) [17] and regular stacked autoencoders (SAE). Moreover, the classification results provided by SDA were then compared to SAE, Support Vector Machines (SVM) [18], Random Forest (RF) [19], and regular autoencoders. The work proposed in [20] shows a novel approach of creating a new type of autoencoders, in which it combines stacked autoencoders and LSTM network. The work was separated into two-phases—(1) feature transformation using the LSTM-stacked autoencoders, and (2) applying LSTM for fault identification. The proposed method focused on detecting injected component faults to a Bently Nevada Rotor Kit RK3, which was designed to physically simulate rotating equipment and its conditions. The raw vibrational signals were directly collected from the RK3 kit, then the Wavelet Packet Decomposition (WPD) method was used to select features in both the time-domain and the frequency-domain, to ensure a wide investigation in both domains, followed by transforming the selected features using the stacked autoencoders in account to their mean square error calculated, which helped to generate a threshold for each feature. Finally, the fault detection accuracy for each feature was validated using a five-fold cross validation, after classification using the K-Nearest Neighbor (KNN) [21] method. No comparisons of other feature selection methods to WPD, or additional classifiers besides KNN were used in the mentioned work. According to [22], a component fault diagnosis system of rolling bearings using stacked autoencoders was introduced and compared to two other deep learning schemas—(1) deep Boltzmann machines and (2) deep belief networks. Four experiments were conducted using various data pre-processing schemas using the time-domain, frequency-domain, and the time-frequency domain.

As stated in [23], a deep autoencoder was developed to diagnose vibration signals in both gearboxes and electrical locomotive roller bearings. The novel approach proposed consisted of two steps—(1) the design of the deep loss function in the autoencoder using maximum correntropy, and (2) applying the artificial fish swam algorithm to optimize the autoencoder’s parameters and its ability to extract valuable features. Similarly, the approach proposed in [24] demonstrates a new method of combining wavelet transform and stacked autoencoders, to diagnose faults occurring in the roller bearing systems. Furthermore, a deep autoencoder in [25] was used to develop the quality of feature fusion, which contributes to aiding the diagnosis of faults in rotating machinery. The applied autoencoder was a collaboration between denoising autoencoders and contractive autoencoders. Where the deeply extracted features from both methods were then separately fused together using locality preserving projection (LPP). The fused features were then applied to the SoftMax function, to train the diagnosis process. In addition, another architecture of sparse autoencoders was performed in [26] to monitor and diagnose the component faults in motors and air compressors. The application of regular ML classifiers, such as SVM, requires intensive understanding and expertise in feature engineering. Thus, the application of autoencoders can massively facilitate the feature engineering process and perhaps outperform the regular feature engineering approaches. For that matter, sparse autoencoders are compared to other ML fault diagnosis methods, such as SVM and SoftMax regressor, to classify faults in motors and air compressors. Accordingly, in [27], a multivariant fault diagnosis and health monitoring approach in rotating machines is introduced. This method is called “SAE-DBN”, as a combination of a two-layered sparse autoencoder (SAE), to perform data fusion between the features of multi-sensors, followed by the application of Deep Belief Networks (DBN) for diagnosis. In [28], another approach using sparse autoencoders is proposed. The method is applied to induction motors monitoring and fault diagnosis purposes. The autoencoder application in [29] shows an ensemble, and deep approach of autoencoders designated to fault diagnosis in rolling bearings. Various activation functions are deployed at the same time, to create multiple autoencoders that are going to be combined later, using a novel strategy. Finally, the work in [30] investigates fault detection and feature extraction schema for motors, using an autoencoding schema of Recurrent Neural Networks (RNN). The explained schema for fault classification is applied directly on time-domain vibrational data and then compared to the results conducted by a two-layered Artificial Neural Network (ANN) [31] model. On a different note, the feature selection capacities of the RNN autoencoder was compared to PCA and Linear Discriminant Analysis (LDA) [32] for dimensionality reduction. The vibrational signals used in this work were obtained from an actual motor positioned with different accelerometers in various locations. The Table 1 demonstrated below is created to conclude all autoencoding FDD approaches in mechanical machinery that was performed in the past decade.

## 3. Hydraulic System FDD Overview

In this experiment, the data used were collected from a condition monitoring of a hydraulic test rig, which was designated to test a hydraulic system. Thereafter, the data were used to conduct two main experiments, one to analyze the provided component faults at the total failure stage. The other experiment was concerned with sensor faults, where the fault injection took place to successfully inject three main types of sensor faults; constant fault that covers constant low, constant high and constant zero faults, as well as gain faults and bias or offset faults. These injected faults along with the healthy readings would eventually act as pre-defined classes necessary for fault classification and healthy signal reconstruction learning, respectively.

**Figure 1:**
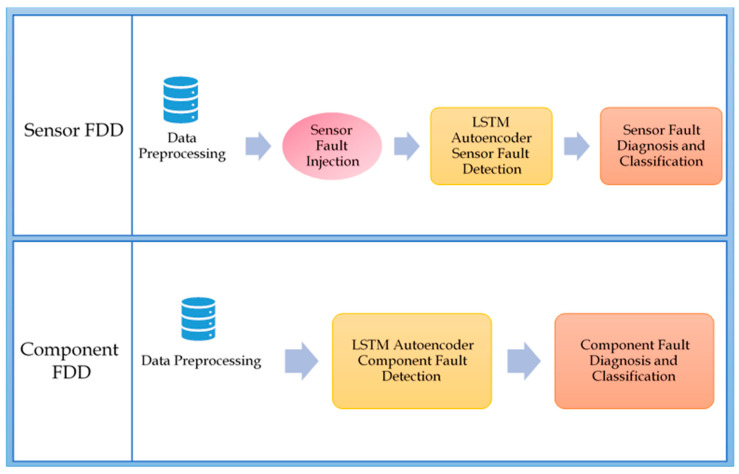
An Overview of the Two Experiments to Achieve FDD in Hydraulic Test Rigs for both Sensor and Component Faults.

Figure 1 shows the two main experiments to detect and diagnose a variety of sensor faults and severe component failures in the hydraulic test rig readings, as an example of a hydraulic system’s data. In this work, two comprehensive experiments were conducted to guarantee performing fault detection and diagnosis for each component and sensor faults in the hydraulic system tested, using a hydraulic test rig. The two experiments start mutually with applying the necessary data pre-processing steps, to ensure removal of unnecessary noise in the input signals, and to arrange the inputs in a way suitable for the LSTM autoencoder. Please note that the data applied, and its pre-processing differed between the sensor FDD experiment and the component one. Moreover, the data used for both experiments were filtered and organized differently. The data description and organization for both experiments are described in detail in the experimental results section of this work. For Sensor FDD, the available dataset of a hydraulic test rig lacked the presence of any sensor faults, which necessitated the injection of various sensor faults into the filtered and pre-processed data. The choice of which types of sensors faults to inject was decided upon convenience and necessity. For example, stuck-at or constant sensor fault was chosen to be injected in the data due to its simplicity to apply such an effect on different periods of time, as compared to other data-centric sensor faults, i.e., outliers or spikes that were not easily predicted or occurred frequently, or even possessed a regular pattern in which they could be injected in the data. Gain and bias faults are an example of system-centric faults, which by definition are complicated to diagnose relying on the data alone. This is why these sensor faults are significant to study and apply algorithms with high accuracies to diagnose. Both mentioned faults also had a clear definition and pattern that made it easier to inject them to the dataset.

For the component FDD experiments, the instances selected were the ones with full efficiency, to be fed in the detection phase demonstrated by the LSTM autoencoder. However, the faults that proceeded to the diagnosis stage were the ones representing total failure in the hydraulic test rig, which were, cooler total failure, valve total failure, pump severe leakage, and hydraulic accumulator total failure. In both experiments, the detection phase was demonstrated by the LSTM autoencoder to reconstruct the healthy form of the input signal when the FDD was conducted and tested. The LSTM autoencoder was trained using healthy sensor data that showed full efficiency in both experiments. However, the data formulation and organization were different between the two experiments, since the signal subject of reconstruction for sensor FDD was a window of 60 s values that separately corresponded to each sensor in the hydraulic test rig. However, in the component FDD, the healthy signal used for training was organized without sliding windows, while each reading represented the values of all eleven sensors at this particular point of time, and how they altogether contributed to diagnosing the failure.

In the diagnosis phase for both experiments, the faulty data of both systems were separately fed into a classification process. The choice of traditional ML classifiers for this experiment was dependent on the selection of various supervised learning methods of entirely different functionality and mechanism, as possible, to provide a broad and comprehensive analysis. The classifiers used in this experiment were LDA, Logistic Regression (LR), KNN, Decision Trees (CART), Naïve Bayesian (NB), SVM, and finally RF. The DL methods chosen for this experiment were CNN and LSTM, both applied in an interesting manner. The comparison included the application of the chosen ML and DL approaches using different features extracted or selected via numerous feature extraction and selection methods, such as manually extracting time domain features for each sliding window, such as the mean, variance, standard deviation, and signal to noise ratio. PCA was also applied directly using the raw multivariate time domain sensor data without dimensionality reduction, and by using the Recursive *k*-Means Silhouette Elimination (R*k*SE) feature selection and dimensionality reduction algorithm [36]. Finally, the trained models and saved thresholds from each experiment could be easily used to achieve run-time predictions of new samples at real-time. The FDD prediction at run-time could be done by the following. (1) Detection—(a) for predicting the healthy reconstructed sample to the new sample using the trained model of the LSTM autoencoder; (b) comparing the reconstructed sample and its original form by applying the suitable sequence difference; (c) comparing the calculated sequence difference to the threshold computed during the offline training stage (if the difference was greater than the threshold then a fault has been detected; when a fault was detected, it needed to be passed to the next stage of fault diagnosis). (2) Fault diagnosis—this step was done by passing the new sample that was detected as faulty, into the chosen trained classifier. This was done by taking into consideration and choosing the best features and the most optimal classifiers, based on the comprehensive training and comparisons done previously in the model training offline phase.

## 4. Analysis and Experimental Results

### 4.1. Experiment One: Sensor FDD Using the Joint LSTM Autoencoder and Classifier Approach

In this section, FDD of sensor faults in hydraulic test rigs using a joint approach between healthy signal reconstruction to detect sensor faults, followed by fault classification to diagnose the selected sensor faults was introduced, analyzed, and discussed.

**Figure 2:**
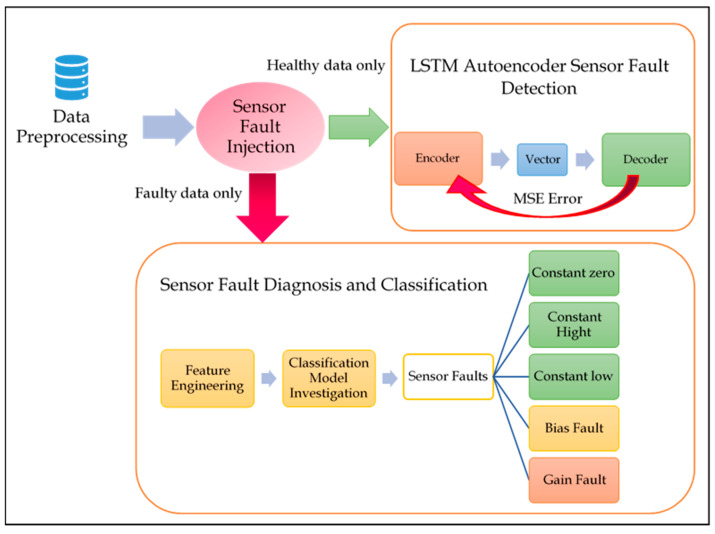
Sensor FDD Comprehensive Framework.

The following subsections elucidate each step of the described approach applied on sensor faults and showcase their results. Figure 2 shows the steps included in experiment one, where each step, stated below, is elaborated in comprehensive detail.

The dataset used for the sensor fault detection and diagnosis was the hydraulic test rig dataset earlier. The mentioned data provided a wide range of component faults that varied from slightly damaged to total failure. However, the dataset did not provide any sensor faults. Thus, it was essential to inject sensor faults to build the sensor FDD model.

Although the sensor FDD architecture navigated in this work was meant for multivariate time-series data, for simplicity, only one sensor only was considered to show results for the sensor FDD process. Sensor PS1 (the first pressure sensor) was used to showcase the sensor FDD results during the fault injection, and sensor FDD was used for the LSTM autoencoder and the sensor detection classification results.

The sensor fault types, their equations, and some of their recent applications are mentioned shortly in [6,37,38,39]. The faults chosen to be injected were as follows. (1) Stuck-at—three main types of stuck at faults was injected, as the stuck-at or constant faults are the most common form of data-centric faults, and it reflects the severity of the sensor condition. Moreover, constant faults are extremely easy to inject. Consider the input sensor signal was x(t), then the constant fault could be easily injected by following $x^{\prime }\left(t\right)=c$, where c is a constant number representing the stationary condition of the sensor. Three main types of constant faults were added. Constant zero when the sensor was stuck at zero, constant high when the sensor was stuck at the highest value in the window, and constant low when stuck at the lowest point of the sensor readings during the observed window. We randomly injected 40 windows of size 60 s (because the sensors in the dataset repeated in a duration of 60 s) with a constant zero fault, 7210 windows of size 60 readings of PS1 was injected with high and low constant faults, which make the overall number of windows injected with stuck-at fault to be 7250 windows. (2) Gain faults; and (3) Bias or offset faults—these faults are a type of system-centric faults; hence it is hard to observe their pattern through sensor signal’s observation alone. Therefore, these faults are significant to study and build ML approaches to dynamically detect and diagnose them. Furthermore, both faults show a clear pattern that makes it easy to inject these types of faults in the data. Gain fault is also known as amplification, where the original signal x(t) was amplified with a constant w; $x^{\prime }\left(t\right)=x\left(t\right)*w$. To inject this fault, a randomly selected amplification number between 0.3–1.3 (equivalent to 30%–130% of the original signal, was applied as gain or multiplication) was selected each time, to regenerate the magnified fault signal. A total of 7210 samples of 60 PS1 sensor readings were injected with randomly chosen gain values. Bias or offset fault was another example of calibration system-centric fault, where the original signal was shifted with a constant value. Consider the original signal to be x(t), then the manipulated offset signal would be $x^{\prime }\left(t\right)=x\left(t\right)+b$, where b is the constant number representing the bias or offset added to the signal. The b value can be too small and hard to notice or observe, or too large and hard to ignore. As a result, it is essential to inject both cases of b. To achieve this, 3480 windows of size 60 were injected with a random number between 0.1–1 to represent the too tiny bias category, while the remaining 3730 windows of size 60 were injected with the comparatively larger biases that were randomly chosen between 1.1–50 (it is not possible to show percentages here, because this is an additive value to the original signal, not a multiplied percentage of it, as in the gain faults). Finally, the overall PS1 sensor data prepared after the fault inject process, possess many windows of size 60 readings that consist of the following—(1) 7210 windows representing fully efficient windows as an example of healthy windows; (2) 7250 windows of constant faults (zero, high, and low); (3) 7120 windows of gain fault; and (4) 7210 windows of bias faults (low and high bias).

#### 4.1.1. LSTM Autoencoder for Sensor Signal Reconstruction

To achieve the problem under investigation, the desired neural network should be able to perform sequence-to-sequence predictions. Hence, the input sequence was the sliding window of the sensor PS1 and the reconstructed signal was from the same nature of the input sequence, and they both had the same size of 60. Then, the encoder–decoder type required to fit the problem was an autoencoder. The choice of LSTM as a type of DL algorithm was due to its tendency to learn the hidden dependencies between many time points at once, which make LSTM one of the most suitable forms of DL when it comes to time-series data, especially sequence-to-sequence (seq2seq) operations.

The LSTM autoencoder created for this experiment, only had one batch of LSTM sequences. This batch was designed to be sequential in direction and nature, which meant that the input layer was directly connected to the hidden layers, then the hidden layer was connected to the output layer. The LSTM hidden layer consisted of a hundred hidden LSTM neurons. The activation function applied for the designed DL model was ReLU, based on a trial and error validation. The hidden layer was chosen to be fully connected by adding the dense layer of output equal to the overall output expected from the LSTM model. The optimizer chosen for the LSTM layer was the Adam optimization algorithm.

In order to utilize the healthy windows of PS1 for LSTM use, it must go under a heavy pre-processing and structuring, to fit the LSTM criteria. The pre-processing and restructuring included the following—(1) flatten the data into a vector; (2) normalize the flattened data between zero and one that can be used in LSTM; and (3) create the target sequence y(t) to reconstruct the most important step of all, which determined what to learn and what to predict. In our case, the input sequence was a sliding window of size 60, while the target sequence was the next sliding window. The shift or sliding step was assumed to be only one step to guarantee a higher model accuracy, which meant that if the input point was x(0), then the target point used to train the prediction model was y(0)=x(1). Therefore, in general, y(t)=x(t+1) (4) divide the flattened normalized vectors of x(t) and y(t) between the training and testing samples, where the training windows were the 80% selected from the overall data, while the remaining 20% was equally divided between testing and prediction. (5) The next step included converting the flat, normalized vectors of x(t) and y(t) into a two-dimensional array (number of samples, window size) shape; followed by (6) converting the training and testing 2D tensor samples into a 3D tensor suitable for use in LSTM. LSTM units in Keras only accept the training and testing data in a 3D tensor shape following the size (number of samples, time points, number of features). Where the z-axis or pages or axis 0 is the number of samples, axis 1 or rows is the number of time-points to store in the memory of LSTM and learn their dependencies, and finally axis 2 or columns represents the number of features inserted in the data. The x(train) used in this experiment were of size (11191, 60, 1), where 11191 of samples were of size (60,1), which corresponded to one window of 60 only healthy readings of PS1.

The previously designed LSTM model was trained and validated using the intensely pre-processed healthy data of PS1. In this experiment, the LSTM parameters were set to one hundred epochs and the verbose equaled one.

The validation results of the LSTM healthy signal reconstruction using the formulated testing data at the last epoch (number one hundred) had the errors Mean Square Error (MSE) and Mean Absolute Error (MAE) 0.000039871 and 0.0029, respectively, which are both considered to be exceedingly small loss values.

After training, evaluating, and testing the LSTM autoencoder model, it was time to start making fault detection decisions aided by the model. However, the question arises as how to detect faults based on the quality of the reconstructed signal? Which brings up another important question—how to determine the fault detection threshold?

Taking a glance at the state-of-the-art methods helps answering the previous questions, e.g., [13]. The approach applied there was similar to ours, as they had separate phases for both detection using signal reconstruction, and diagnosis by applying fault classification. To find the difference between the predicted sequence and the input sequence, they used signal difference that could be easily calculated by taking the amplitude of the subtraction operation between the two sequences ($z\left(t\right)=\left|x\left(t\right)-x^{\prime }\left(t\right)\right|$). Although using signal difference shows accurate results, we propose a different signal similarity measure that showed more accuracy and performance, when compared to signal difference for fault detection.

The threshold determines what is faulty or healthy, based on the value of the signal difference. If the value was higher than the designated threshold, then the reconstructed signal was considered faulty, or else it was healthy. The threshold was best measured by creating a pool of various threshold values between the minimum and maximum values of the calculated signal difference. This was followed with making the fault detection decisions on the prediction samples, based on each threshold in the pool. For each threshold in the pool, we checked if the prediction’s sample signal difference was higher than the threshold that was considered faulty; where a lower signal was detected to be healthy. Finally, the precision, recall, f1-score, and accuracy for all prediction results made by each threshold in the pool were calculated. The choice of the right threshold for the sensor fault detection was made by choosing the threshold that guaranteed the best precision to recall trade-off, also known as the f1-score. In this experiment, a prediction dataset (500 windows of size 60 readings of PS1) that consisted of various healthy and faulty samples, was used to determine the fault detection accuracy using the LSTM autoencoder sequence reconstruction. The 500 windows reconstructed using LSTM were then each compared to the original sequence to show how much they deviated from the original window with regards to their health status. The comparisons between the reconstructed windows and the original ones were made by utilizing two main metrics—(1) signal difference: $z\left(t\right)=\left|x\left(t\right)-x^{\prime }\left(t\right)\right|$; and (2) our new metric that used the complement of Pearson’s autocorrelation.

Pearson’s autocorrelation could be calculated using the formula shown below:

(1) $$r_{xy}=\;\frac{n\sum^{\;}x_{i}y_{i}-\;\sum^{\;}x_{i}\sum^{\;}y_{i}}{\sqrt{n\;\sum^{\;}x_{i}^{2}-{\left(\sum^{\;}x_{i}\right)}^{2}}\sqrt{n\;\sum^{\;}y_{i}^{2}-{\left(\sum^{\;}y_{i}\right)}^{2}}}$$

where $r_{xy}$ is the correlation between vectors x, y. Furthermore, x and y were expected to possess the same length of n. Here the autocorrelation measured the similarity between two sequences, while subtracting the measured similarity from the highest possible value of resemblance (+1) represented another way of calculating the difference between two sequences $z\left(t\right)=1-\;r_{xy}$.

The tables demonstrated below show some of the threshold values selected to find the optimal threshold necessary for the sensor fault detection, corresponding to their precision, recall, f1-score, and accuracy, using the traditional signal difference, and our proposed correlation complement.

Based on the values shown in Table 2 and Table 3, the optimal threshold from each signal difference metric could be easily detected by choosing the threshold that provided the best precision and recall trade-off. It was apparent that the threshold of 0.3 was the optimal threshold when using the regular signal difference metric, and the accuracy of the LSTM autoencoding sensor fault detection when using the optimal threshold of 0.3 was 0.62. On the other hand, the optimal threshold when using the signal difference based on the correlation was 0.5, and the accuracy of the sensor detection given the optimal threshold was 0.71.

**Figure 3:**
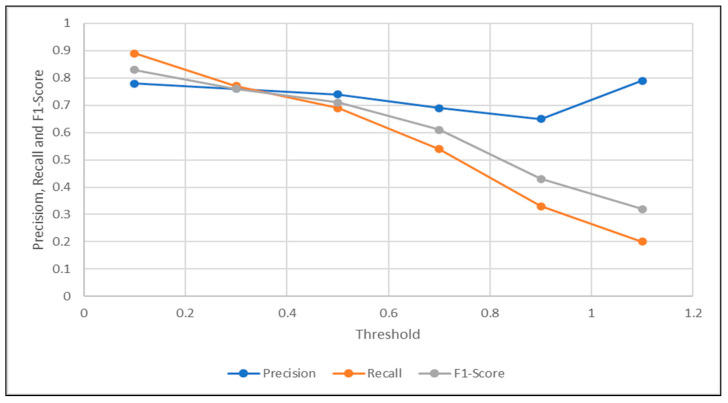
Optimal Threshold Selection Using Regular Signal Difference.

**Figure 4:**
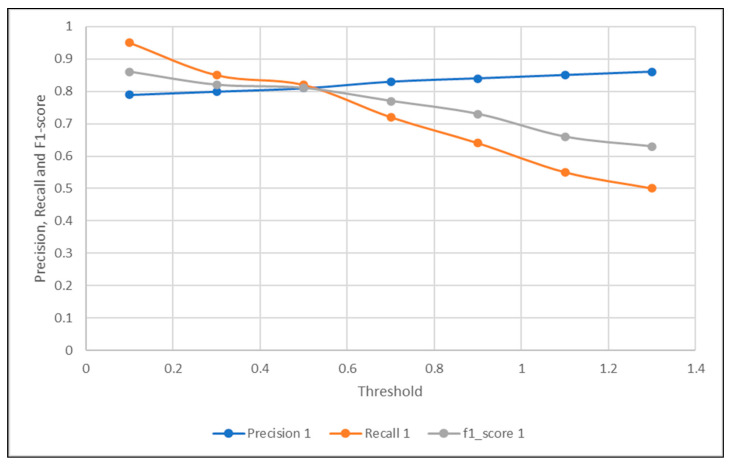
Optimal Threshold Selection Using Signal Difference Based on Correlation Complement.

As visualized in Figure 3 and Figure 4. The optimal threshold provided the best precision and recall trade-off, also known as the f1-score. The optimal threshold could be easily observed as the intersection point between the three metrics mentioned previously. Based on the visualization in Figure 3, the threshold selected was 0.3, which provided the detection with 0.62 accuracy. Compared to the intersection point shown in Figure 4, when the threshold was chosen to be 0.5, the corresponding accuracy was observed to be higher at 0.71. This concluded the accuracy of the proposed signal difference measure as compared to the traditional one, to achieve fault detection using the signal reconstruction technique.

#### 4.1.2. Sensor Fault Diagnosis—Classification Schema

In this section, the experimental results conducting sensor fault diagnosis using a variety of supervised learning algorithms is demonstrated. As shown in Figure 2, the second phase following the detection of existing anomalies involved applying necessary means to diagnose their nature. The faults detected by the previous phase using the LSTM autoencoder, were then fed into a fault classification schema, to determine the type and nature of the detected faults. In other words, to perform the fault diagnosis, only faulty data were classified.

In this work, the classification results were compared when various feature engineering approaches were applied. The feature engineering approaches used for this section were—PCA, feature importance (FI), manually extracted time-domain features, and a new cluster-based feature selection method called R*k*SE. Feature selection or extraction when applied to univariate datasets in the shape of sliding windows, is simply considered to be a window compression method, to minimize the size of the readings provided by each window and select the features with most contribution to the learning process. Therefore, the time and complexity constraints of the ML or DL models could be managed and minimized with a smarter choice of features. Various ML and DL classifiers were individually trained, validated, and tested with the selected features, using a diversity of feature engineering approaches. Then, their results were documented and compared. The ML approaches used were LR, LDA, KNN, CART, NB, SVM, and RF. The DL approaches selected to perform the classification tasks were CNN and LSTM.

The following experimental results tackled each of the feature engineering process and their results when fed into the above-mentioned ML and DL classifiers, to eventually achieve the FDD for sensor faults, using the PS1 sensor as an example of sensors in the hydraulic test rig system.

The parameters selection for each classifier was chosen by trial and error, to ensure the highest possible accuracy when validating using 10-fold cross-validation over the original data, without any feature engineering applied. The table below describes the applied ML classifiers and their corresponding parameters using Scikit in Python. Furthermore, the mean accuracy for the 10-folds and the standard deviation corresponding to the 10-folds was calculated.

The CNN classifier had the parameters verbose, epochs, and batch size of zero, hundred, and twenty, respectively. The parameters were chosen by trial and error to provide the designed deep neural network with the highest 10-fold classification accuracy. The CNN applied was designed as the sequential model (input, hidden layers, and output). The CNN convolutional layer applied here was a 1-D layer, since the training dataset was a time-series data and of a one-dimensional nature, unlike the usual application of CNN where the data are typically of two-dimensional shapes, such as images. The CNN design included 6 one-dimensional convolutional layers of filter, equaling to 64 and a kernel size of one. The kernel size that showed the length of the convolution window/masking window required for the convolution was selected as one. The number of convolution layers was added to guarantee the highest possible accuracy, and through trial and error, it was set to 6 layers of 1-D convolutional layers. The activation function within the created layers was ReLU. The next process following each convolution layer was the pooling layer. In this work, the pooling function was selected as the maximum pooling, which indicated selecting the maximum entry in the kernel during the pooling phase. Two fully connected layers were added, following the pooling phase, one of size hundred and their activation function was ReLU. The second fully connected layer had three outputs to match the number of classification outputs/faults designated for the training, while its activation function was selected as SoftMax. Finally, the CNN optimizer chosen was the Adam optimizer. The LSTM model designed for classification differed from the one used in the previous step, as this model was a classifier while the previous LSTM model was an autoencoder designed to solve multi-regression problems and not classification. Only one batch of LSTM neurons was used, this batch had hundred sequential hidden layers or neurons. The layers were fully connected using a dense layer of size one hundred with the activation function ReLU, which was connected to another fully connected dense layer of size three (to match the number of outputs expected from the LSTM model), with the activation function SoftMax. The LSTM classifier parameters were represented by verbose, epochs, and batch size, which were equal to 0, 10, and 20, respectively.

The classification results for ML and DL classifiers when fed with only faulty data, to perform PS1 fault diagnosis and classification are listed. Table 4 lists nine ML and DL classifiers that were trained separately using five different features at a time, which were selected/extracted using PCA, manually extracted time-domain features, FI, R*k*SE, as well as the entire faulty dataset without any feature selection. The number of features without feature selection was equivalent to the window size of 60. Four features were extracted using PCA, 45 features were selected using FI, compared to 46 features selected using R*k*SE. Finally, four time-domain features extracted from each window are represented by the mean, variance, standard deviation, and signal to noise ratio. The number of features selected by each method was the one with the highest fault classification mean accuracy.

When observing each row with respect to each feature engineering method, the feature engineering approach giving the highest or lowest 10-fold mean accuracy corresponding to each classification method are clearly shown. The mean feature accuracy row shows the overall accuracy for each feature engineering approach, with respect to all ML and DL classifiers combined. PCA had the highest mean feature engineering accuracy when applied to the nine classifiers, which proved the consistency of PCA and its validity with different classification techniques. It was also obvious that the time-domain features selected were the one providing highest accuracy to some ML and DL classifiers, which were LDA, KNN, CART, RF, and LSTM. However, this feature selection technique did not provide consistency in the accuracy results, since the LR and NB classifiers showed exceptionally low performances when applying the four selected time-domain features, as compared to the rest of the feature engineering methods. This explains why the time-domain features result in lower overall mean feature accuracy, as compared to PCA, even though more classifiers have the maximum accuracy when applying time-domain features.

The selection of the suitable feature engineering method was highly dependent on each classifier type and its functionality. The table above serves the purpose of investigating the behavioral changes of some of the most common ML and DL classifiers, with respect to various commonly used feature engineering methods with time-series datasets. Furthermore, finding the best pair of features and classification approach that provides the most optimal accuracy–complexity trade-off when performing sensor fault detection, is the number one aim of these comparisons. As a result, the highest measured sensor fault detection combination was when CART was applied using time-domain features, followed by LSTM, KNN, and RF, using the same extracted features.

### 4.2. Experiment Two: Component FDD Using the Joint LSTM Autoencoder and Classifier Approach

**Figure 5:**
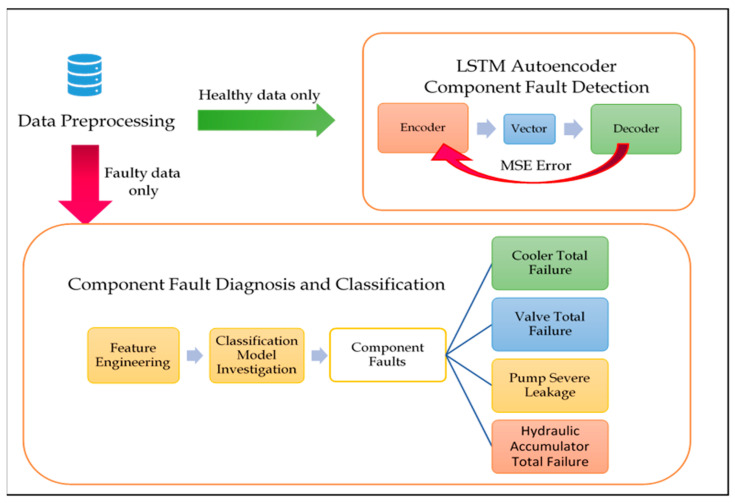
Component FDD Comprehensive Framework.

In this experiment, the component faults existing in the hydraulic test rig were detected and diagnosed using a unique approach, in which the detection and diagnosis stages were carried out separately to ensure more accurate detection of rare occurrences. Figure 5 shows the framework of this experiment.

The same steps and parameters created in experiment one (sensor FDD) were repeated in this experiment, excluding the data pre-processing and structuring, and the fault injection schema. The pre-processing step differed from the previous experiment, since this time it was a multi-variate autoencoding and classification problem, without the application of sliding windows. Moreover, no fault injection was required in this experiment because the component faults studied were already available in the hydraulic test rig dataset used for this experiment. In this section, the data used were the hydraulic test rig dataset of eleven sensors, which indicated that this was a multi-variate FDD experiment. The healthy data were applied for detection as the first step of the FDD system, represented by the LSTM autoencoder. While the faulty data contained four main component faults—cooler, value, hydraulic accumulator total failures, and pump severe leakage fault, were used in the second stage, which was represented by the fault diagnosis, using the supervised ML and DL methods.

In both stages, the data were organized as a 2D matrix of samples and features, expressed by the eleven sensors and their readings, at different time-points.

#### 4.2.1. Component Fault Detection—LSTM Autoencoder

This section had the same procedure as that explained in experiment one for fault detection in sensors. The LSTM autoencoder for fault detection stage had the following main steps. (1) Design the LSTM autoencoder to fit the problem. (2) Prepare the data into a form acceptable in the LSTM. (3) Train and validate the LSTM autoencoder using only healthy data and calculate the MSE and other error metrics. (4) Predict the samples that contain the faulty and healthy readings. (5) Calculate the signal difference between the original samples and the predicted samples, using the regular difference and the Pearson’s correlation, to establish accuracy comparisons. (6) Find the best threshold of sequence difference to ensure the best trade-off between precision, recall, and f1-score. (7) Make component fault detection decisions using the trained, validated LSTM autoencoding model, and their calculated sequence difference, as compared to the computed threshold.

For training the designed model, 1438 samples of the eleven sensors’ reading were used to train and validate the model. The data should be normalized between zero and one, as well as converted to a three-dimensional tensor format (samples, time-points for LSTM to remember, number of features), before applying to the LSTM model. The LSTM model designed for component FDD detection was an autoencoder of a sequential hundred hidden LSTM neurons, using the activation function ReLU, then a fully connected dense layer of size equal to the number of sensors or features was added. The dense layer contributed to improving the accuracy of the LSTM model, as well as making sure the LSTM model generated outcomes equal to the designated input signal, in terms of size. Finally, the optimizer applied for the LSTM model was Adam. The training parameters of the LSTM autoencoder were epochs = 100 and batch size = 30.

After training and validating the designed model over a hundred epochs, the MSE error of the last epoch was 0.00057 and the MAE was equal to 0.0096. Both error metrics were exceedingly small, which was a high indication of the validity and accuracy of the created model in reconstructing healthy input sequences.

To select the optimal threshold corresponding to the allowed sequence difference between the original sequence and the one reconstructed from the LSTM autoencoder, 4800 samples of size eleven were predicted using the autoencoder, to reconstruct 4800 healthy versions of the prediction samples. The signal difference between each of the corresponding sequences in the original and reconstructed sequences were computed using (1) The traditional signal subtraction to find the signal difference as a vector, and then to find the magnitude of this vector. (2) The sequence difference using (1-Pearson’s autocorrelation) as a measurement created in this work is proposed to be a more accurate measurement for fault detection than the traditional signal subtraction.

To avoid repeating the explanation of each signal difference methods, we jump right through the results and their comparisons.

**Figure 6:**
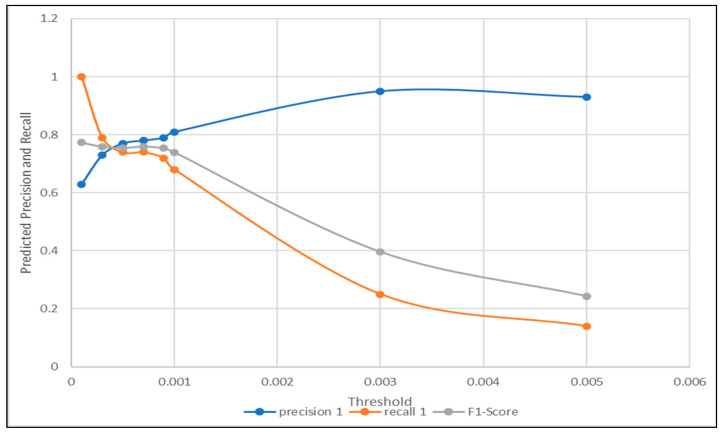
Precision, Recall, and f1-Score trade-off for Threshold selection using Pearson’s Correlation Difference.

A pool of candidate threshold values was created, then the labels of the 4800 prediction samples were obtained based on each threshold in the pool, if the value of the signal difference was higher than the threshold a fault was considered to be detected, thus receiving a label 1, else label 0. The precision, recall, and f1-score were computed for each threshold in the pool, based on the generated labels and the original labels of the given prediction samples. When applying signal difference using the Pearson’s autocorrelation complement, the pool of chosen thresholds between the minimum and maximum observed values are shown in Table 5. Furthermore, for each chosen threshold, the accuracy, precision, recall, and f1-score were computed. Figure 6 illustrates the process of choosing the component fault detection threshold based on the precision, recall, and f1-score trade-off shown in the table below. As clearly shown in Figure 6, the selected threshold was the intersection between the three curves, which was approximately equal 0.0007 and the accuracy observed for this threshold value was 0.71.

**Figure 7:**
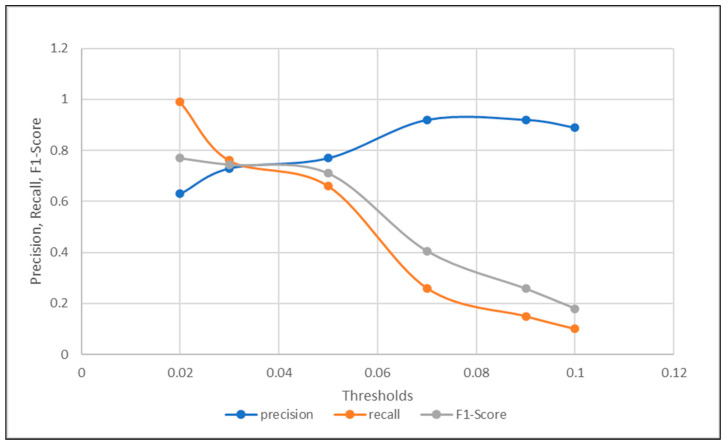
Precision, Recall, and f1-Score trade-off for Threshold Selection Using Signal Subtraction Difference.

On the other hand, the optimal threshold was also calculated when the optimal signal difference was applied. In Figure 7, the intersection between the three curves is demonstrated. It is clearly shown that the optimal threshold for component fault detection using the sequence subtraction, was approximately 0.03, with a fault detection accuracy of 0.69. The optimal accuracy using signal subtraction of 0.69 was less than the measured one using the optimal threshold computed by a Pearson’s correlation of 0.71. As a result, when comparing the accuracy of the optimal thresholds selected using two different signal deviation measurements, which were the autocorrelation complement and the traditional subtraction, it could be seen that the proposed method using Pearson’s correlation complement guaranteed a higher component and sensor fault detection accuracies, as compared to its commonly used traditional subtraction counterpart, based on the measured comparisons in experiment one and two.

#### 4.2.2. Component Fault Diagnosis—Classification Schema

In this section, the feature engineering methods compared were FI, PCA, and R*k*SE. The time-domain extracted features applied for the multi-variate time-series sequences without the application of sliding windows, were expected to have lower accuracy values, regardless of the ML or DL classifier used. Hence, it did not make sense to compute the mean, standard deviation, and variance to a sample of readings extracted from sensors of different nature. However, the time-domain features were extracted and applied to all classifiers, to prove the point mentioned earlier.

The optimal number for each feature engineering method was computed for each method to ensure the best accuracy and complexity trade-off. The overall number of features in each sample was eleven, corresponding to each sensor in the hydraulic test rig, the optimal features for FI, PCA, the time-domain features, and RKSE were four, five, four, and nine, respectively.

The accuracies computed for all ML and DL classifiers were the results of dividing the fault data with component faults, into training and testing data, with percentages of 80% to 20% of the faulty data, respectively. Followed by applying a 10-fold cross validation technique for each classifier separately.

The parameters and optimizers for each ML method used in this section, were identical to the ones used in the sensor FDD experiment earlier in this paper. Moreover, some minor changes in the CNN and LSTM classifiers’ design and parameters was made from the previous experiment.

The CNN design consisted of only one 1D CNN layer of filter size 64, and kernel size of one. The layer was sequential which meant that the input layer was directly connected to the hidden layer(s) that was connected to the output layer. The activation function used was ReLU. Followed by the pooling layer that had a pooling size of one, maximum pooling was applied as the pooling function. Finally, a fully connected dense layer of size equivalent to the number of expected outputs, with the SoftMax activation function was created to make the classification process for the extracted features during the convolutional and pooling layers. The CNN optimizer used was Adam, as a stochastic gradient descent approach to optimize the network. The LSTM classifier applied had only one LSTM batch with two hundred hidden neurons that were sequential in order and nature. Followed by a fully connected dense layer of SoftMax activation function, Adam was the applied optimizer for the LSTM as well. The verbose, epochs, and batch size parameters were applied by testing various values and their effect on the classification accuracy, and they were set to zero, 10, and 20, accordingly.

The table below comprehend the component fault classification results when trained by faulty hydraulic test rig data, applying various ML and DL approaches using numerous feature engineering methods.

As shown in Table 6, the feature selection approaches worked better than the extraction ones, such as, PCA and time-domain features, when dealing with traditional ML classifiers, to classify multi-variate time-series datasets. FI consistently showed the highest accuracy results as compared to the rest of the feature engineering methods when dealing with traditional ML classifiers. This was followed by R*k*SE, which had a slightly lower accuracy than FI for traditional ML approaches, but showed consistency in all ML classifiers. Moreover, PCA showed the highest accuracy when applying DL classification algorithms, as compared to other feature selection approaches. FI and R*k*SE were neck-to-neck when it came to the classification accuracy using the selected DL approaches. The time-domain features were the most accurate ones when applied to sliding-windows for univariate classification, as shown in experiment one. However, as spotted earlier in this experiment, in terms of extracting time-domain features from multi-variate datasets without applying sliding windows, this feature extraction method proved to be weaker than the rest of the approaches, when combined, irrespective of the ML or DL classifiers.

In comparison, KNN, CART, RF, and SVM showed a greater consistency in high accuracy results irrespective of the feature engineering method applied, including time-domain features irrespective of its weakness. CNN and LSTM had lower accuracies as compared to the traditional ML approaches mentioned earlier, and their accuracies dropped radically when the time-domain features were applied.

To conclude experiment two, it was important to know how to apply the trained models and saved parameters from experiment two at run-time, to make new real-time predictions. The input vector for prediction should have one readings of each sensor of the eleven sensors used for the training process, during the offline or training phase. (1) Fault detection—fault detection for new samples could be done by feeding the new sample into the trained and validated LSTM autoencoder model, to reconstruct the healthy form of the sequence. Then, the reconstructed sequence and the original one was compared by calculating the signal difference using Pearson’s autocorrelation. Finally, when the signal difference calculated was above or below the trained threshold, this would determine the existence of faults. (2) Fault diagnosis—in case a fault was detected using the detection step, the fault should be diagnosed by applying the necessary feature engineering approaches, then the new processed sample should be fed to the highest accuracy ML or DL trained classifier, suitable to the features chosen. In our case, based on the results shown in Table 6, it was more accurate to use RF combined with FI to guarantee better accuracy and complexity trade-off.

## 5. Conclusion and Discussion

In this section, a two-staged FDD approach is proposed. Where the detection and diagnosis are separated into two stages to guarantee detecting rare occurrences and events in hydraulic systems. The detection process is represented by a LSTM autoencoder that learns only from healthy observations, in an attempt to reconstruct the healthy version of the given sequences, sensor window readings, or multi-sensors readings. This is followed by the comparison between the given sequences and their healthy reconstructed version, to measure the deviation from the healthy state and the given sequences, which is vital to detect the existence of faults and malfunctions when this deviation exceeds a certain, learned threshold. The fault diagnosis was represented by a classification process that could be an ML or DL algorithm, which was trained using only the faulty observations captured by the detection stage, using an LSTM autoencoder.

The proposed approach is beyond the state-of-the-art methods, in the following ways:

- The proposed method is applied into two entirely different experiments, with different data pre-processing, acquisition, and structuring; different DL algorithmic designs; and above all for detecting two different fault types—sensor faults and component faults. The methods proposed in the literature only focused on one fault type, either component faults or sensor faults. However, it was rarely seen that any work showed comprehension in detecting or diagnosing different fault types at once.
- In the detection phase, using the LSTM autoencoder, some changes were made from the existing related work. The most important addition was using the sequence difference calculated by subtracting the Pearson’s autocorrelation from one. The detection results using the complement of Pearson’s autocorrelation comparing to the traditional signal difference measure applied in the state-of-the-art research was experimentally proved. In experiment one, to detect sensor faults, the detection accuracy using signal difference was observed as 62%. As compared to the detection accuracy, when applying our proposed measurement of signal difference, the detection accuracy was observed as 71%. Moreover, in experiment two for component fault detection, the accuracies observed using traditional signal difference and the proposed one were 69% and 71%, respectively. The results of the detection phase in the two conducted experiments proved to be the superior of the proposed signal difference measurement, as compared to the traditional subtraction of signals, to provide signal difference.
- Various feature engineering approaches were investigated and paired with numerous ML and DL methods in the diagnosis phase, to determine the most suitable feature engineering method, to classifiers of different functionality and design. Furthermore, this pairing gave the opportunity to see how each classifier reacted with different feature engineering methods of different procedure, which would help future researchers to select the best match pair or avoid the worst pair for both data structures, windows univariate, or no window multi-variate. For example, in experiment one when dealing with sensor faults in a sliding window data structure, it could be seen that the chosen time-domain features showed the highest diagnosis accuracy of almost all classifiers, i.e., LDA, KNN, CART, RF, and LSTM. The mean accuracy of all classifiers using PCA was computed to be a maximum of 82%, which was justified by the consistency PCA shows with all the classifiers regardless their functionality. However, time-domain extracted features showed extremely low diagnosis accuracies when applied to some classifiers, such as LR and NB, with the detection accuracies of only 24.25% and 48.21%, respectively. This explains why the time-domain extraction technique did not have the highest mean accuracy, even though it provided the highest accuracy to the majority of the supervised methods. On the other hand, in experiment two when the component faults were classified using multi-variate sensor readings without the application of sliding windows, FI showed the highest diagnosis accuracy for all ML classifiers, and PCA showed the highest diagnosis accuracy when combined with DL, such as CNN and LSTM.
- In the related work, the diagnosis phase was represented by some chosen type of classifiers combined with a chosen set of features, without any analysis or investigation with respect to other classifiers or features. In this work, after careful experimental observations and calculations, the appropriate features and their suitable classifier was used to represent the diagnosis phase for our algorithm. In experiment one (univariate sliding window structure), the diagnosis phase was chosen using the time-domain extracted features combined with either CART or LSTM classifiers, with diagnosis accuracies of 99.51% and 96.84%. However, in experiment two, when dealing with multi-variate features without the application of sliding windows, FI combined with almost all ML classifiers showed extremely high accuracies exceeding 98%. Thus, RF combined with FI was the best combo used to perform multi-variate diagnosis, especially when FI could be done implicitly during the RF training stage, based on the FI nature, which could help in reducing time and computational complexities.

In conclusion, the proposed approach was used for the first time in the field of industry 4.0, especially when applied to hydraulic test rigs. Although, the proposed work in this paper had multiple changes and valuable improvements from the state-of-the art, there is always a place for improvements and further expansions. For future work, it could be a good challenge to improve experiment one, by designing a LSTM autoencoder that can learn multiple sequences of multiple windows that belongs to different sensors at a time, by applying one LSTM autoencoding model. For example, in experiment one, only one sensor at a time (PS1) was used in a sliding window format to train the LSTM autoencoder to predict/reconstruct the healthy window of the given PS1 window of size 60. In the current approach, we must train the LSTM autoencoder for each sensor separately, to learn how to reconstruct each healthy window. However, a multi-variate approach, multi-sequences, multi-sensor deep neural network design would be a sophisticated approach in the future, where several LSTM autoencoder batches could be connected together sequentially or in parallel, so each could train on different sensor window sequences. Furthermore, the feature engineering methods applied to the diagnosis phase were all in time-domain. Investigating the frequency-domain or the combination of time and frequency domains, such as applying Wavelet Coefficient Packer Decomposition (WPD) would add different perspective for the future of FDD in mechanical equipment.

## Figures and Tables

**Table 1:** Autoencoding-based Methods for FDD in Hydraulic Machinery.

Reference	Autoencoding Method	Mechanical Equipment	Fault Type/Purpose	Dataset
[13]	LSTM Autoencoder + LSTM Classifier	Chemical Reactor	Component Faults of Tennessee Eastman benchmark.	Tennessee Eastman benchmark [14].
[20]	Stacked Autoencoder LSTM + KNN	Rotating equipment	Injected Component faults to a physical simulation	Data collected from Bently Nevada Rotor Kit RK3 to simulate rotating device.
[16]	Stacked Denoised Autoencoder	Rotary machinery	Component faults in a bearing test-rig	Data extracted from physical bearing test-rig.
[22]	Stacked deep autoencoders	Rolling bearings	Component faults in rolling bearings.	Gathered from UPS.
[23]	Another architecture deep autoencoder	Gearboxes and electrical locomotive roller bearings	Component faults in rolling bearings and electrical locomotive.	From a physical test rig.
[24]	Wavelet transform + stacked autoencoders	Roller bearing systems	Component faults in rolling bearings.	From case western reserve university (CWRU).
[25]	Another architecture of deep autoencoders	Rotating machinery	Component faults in rotating machinery	Physical rotor fault test, CWRU [33] and NASA datasets [34].
[26]	Another architecture of sparse autoencoders	Motors and air compressors	Component faults in motors and air compressors	Actual air compressor and motor
[27]	SAE-DBN (sparse autoencoder + Deep Belief Networks)	Rotating machines	Component faults in rotating machinery	Extracted from an experimental system.
[28]	Another architecture of sparse autoencoders	Induction motors	Component faults in induction motors	Fault simulator.
[29]	Ensemble deep autoencoder	Rolling bearings	Component fault diagnosis in rolling bearings	CWRU [33].
[35]	Another architecture of stacked autoencoders	Hydraulic pumps	Detect component faults in hydraulic pumps	Hydraulic pump of type axial piston pump (25MCY14-1B).
[30]	Autoencoding schema of RNN networks	Motors	Component fault detection and feature extraction in motors	Physical motor.

**Table 2:** Signal Difference Thresholds and Their Metrics.

Threshold	0.1	0.3	0.5	0.7	0.9	1.1
Precision	0.78	0.76	0.74	0.69	0.65	0.79
Recall	0.89	0.77	0.69	0.54	0.33	0.2
F1-Score	0.83	0.76	0.71	0.61	0.43	0.32
Accuracy	0.71	0.62	0.55	0.44	0.32	0.32

**Table 3:** Signal Difference using the Correlation and Their Metrics.

Threshold	0.1	0.3	0.5	0.7	0.9	1.1	1.3
Precision	0.79	0.8	0.81	0.83	0.84	0.85	0.86
Recall	0.95	0.85	0.82	0.72	0.64	0.55	0.5
f1-score	0.86	0.82	0.81	0.77	0.73	0.66	0.63
Accuracy	0.76	0.71	0.7	0.66	0.61	0.56	0.53

**Table 4:** Classification Accuracy for Different ML and DL approaches using various Feature Engineering Methods.

Classifier	No Feature Selection	PCA	Time-Domain Features	FI	R*k*SE
LR	0.6911	0.6356	0.2425	0.7019	0.6882
LDA	0.7053	0.6535	0.7859	0.7038	0.7068
KNN	0.8747	0.9128	0.9625	0.8758	0.8816
CART	0.8972	0.9818	0.9951	0.9126	0.9116
NB	0.7089	0.6919	0.4821	0.6930	0.6824
SVM	0.9125	0.8827	0.7298	0.9112	0.9142
RF	0.8189	0.8390	0.9402	0.8196	0.8193
CNN	0.8773	0.8486	0.7575	0.8385	0.8562
LSTM	0.8352	0.9568	0.9684	0.7278	0.7499
Mean Feature Accuracy	0.81	0.82	0.76	0.80	0.80

**Table 5:** The Thresholds of Pearson’s Correlation Difference and Their Corresponding Fault Detection Accuracy, Precision, Recall, and F1-Score.

Threshold	0.0001	0.0003	0.0005	0.0007	0.0009	0.001	0.003	0.005
precision	0.63	0.73	0.77	0.78	0.79	0.81	0.95	0.93
recall	1	0.79	0.74	0.74	0.72	0.68	0.25	0.14
F1-Score	0.77	0.76	0.75	0.76	0.75	0.74	0.39	0.24
Accuracy	0.63	0.69	0.7	0.71	0.7	0.69	0.52	0.46

**Table 6:** Component Fault Diagnosis Using Various Feature Engineering and Classification Approaches.

Method Name	FI	PCA	R*k*SE	Time-Domain Features	No Feature Selection
LR	0.9962	0.7300	0.7823	0.37599	0.6832
LDA	0.7634	0.7490	0.7528	0.370521	0.7031
KNN	0.9940	0.9229	0.9320	0.831458	0.8677
CART	0.9932	0.9435	0.9912	0.928594	0.6849
NB	0.9924	0.7510	0.7122	0.39526	0.9035
SVM	0.9859	0.9337	0.9310	0.833281	0.8139
RF	0.9930	0.9013	0.9910	0.871042	0.9042
CNN	0.7343	0.8427	0.7343	0.3971	0.7385
LSTM	0.7375	0.8770	0.7375	0.3981	0.73124
MEAN ACCURACY	0.910	0.850	0.840	0.600	0.781
